# Assessing the impact of the COVID-19 pandemic on the mental health–related hospitalization rate of youth in Canada: an interrupted time series analysis

**DOI:** 10.24095/hpcdp.44.10.02

**Published:** 2024-10

**Authors:** Christoffer Dharma, Ahmed A. Al-Jaishi, Erin Collins, Christa Orchard, Nana Amankwah, Justin J. Lang, Ian Colman, Murray Weeks, Rojiemiahd Edjoc

**Affiliations:** 1 Centre for Surveillance and Applied Research, Public Health Agency of Canada, Ottawa, Ontario, Canada; 2 Dalla Lana School of Public Health, University of Toronto, Toronto, Ontario, Canada; 3 School of Epidemiology and Public Health, University of Ottawa, Ottawa, Ontario, Canada; 4 Institute for Work & Health, Toronto, Ontario, Canada; 5 Mental Health and Addictions Research Program, ICES, Toronto, Ontario, Canada; 6 Alliance for Research in Exercise, Nutrition and Activity (ARENA), University of South Australia, Adelaide, South Australia, Australia

**Keywords:** mental health, self-harm, substance use–related disorders, youth, adolescents, time series, COVID-19, Canada

## Abstract

**Introduction:**

This study evaluated the effect of the COVID-19 pandemic on temporal trends in mental health and addiction–related inpatient hospitalization rates among youth (aged 10–17 years) in Canadian provinces and territories (excluding Quebec) from 1April 2018 to 5 March 2022.

**Methods:**

We conducted an interrupted time series analysis across three periods: T0 (pre-pandemic: 1 April 2018 to 15 March 2020); T1 (early pandemic: 15 March 2020 to 5July 2020); and T2 (later pandemic: 6 July 2020 to 5 March 2022).

**Results:**

Pre-pandemic mental health and addiction–related hospitalization rates had significant regional variability, with weekly rates from 6.27 to 85.59 events per 100000 persons in Manitoba and the territories combined, respectively. During T1, the national (excluding Quebec) weekly hospitalization rate decreased from a pre-pandemic level of 12.82 (95% CI: 12.14 to 13.50) to 5.11 (95% CI: 3.80 to 6.41) events per 100 000 persons. There was no statistically significant change in the mental health and addiction–related hospitalization rate across provinces and territories in T2 compared to T0. However, there was a significant increase in the rate of self-harm–related hospitalizations among females Canada-wide and in most provinces during this period.

**Conclusion:**

Although several Canadian studies have reported increases in mental health and addiction–related outpatient and emergency department visits among youth during the COVID-19 pandemic, this did not correspond to an increase in the inpatient hospital burden, with the notable exception of self-harm among young females.

**Highlights**

- Before the COVID-19 pandemic, hospitalization rates related to mental health and addiction among youth aged 10 to 17 years varied significantly across Canada, with weekly rates from 6.27 events per 100 000 people in Manitoba to 85.59 events per 100 000 people in the territories.
- The national weekly hospitalization rate (excluding Quebec) decreased from a pre-pandemic level of 12.82 (95% CI: 12.14 to 13.50) to 5.11 (95% CI: 3.80 to 6.41) events per 100 000 persons during the early pandemic period (15 March 2020 to 5 July 2020).
- No significant change in mental health and addiction–related hospitalization rates was noted in the later pandemic period compared to pre-pandemic levels.
- Notably, there was a concerning rise in hospitalization rates for selfharm among female youth across Canada and in most provinces later during the pandemic period.

## Introduction

Social isolation as a result of lockdowns and other societal changes during the COVID-19 pandemic led to a significant increase in mental health disorders such as anxiety, depression and post-traumatic stress disorder, resulting in higher outpatient and emergency department mental health–related visits for youths (<18years).^1^-^6^ Loneliness, anxiety and depression have been on the rise in this population, especially among young females.^7^-^9^ In 2020, females aged 15 to 17 years were twice as likely to be hospitalized for mental health–related disorders than their male counterparts.^10^

Studies focusing on changes in mental health–related emergency department visits and health service utilization among Canadian youths have been conducted in British Columbia, Ontario and Montral (Quebec).^2^,^6^,^11^-^15^ However, there is a lack of research on national and provincial/territorial trends in mental health–related inpatient hospitalizations. With different provincial and territorial public health responses to the pandemic, there may be differences in magnitudes, trends and associations in regional mental health–related hospitalization rates.

Our aim was to assess inpatient hospitalization rates for mental health and addiction diagnoses among Canadian youth across three distinct periods: pre-pandemic (T0), early pandemic (T1) and later pandemic (T2). Focusing on Canadian provinces and territories, excluding Quebec, we provide a detailed analysis, stratified by sex, for youth aged 10 to 17 years. It is important to note that our analysis is specific to inpatient hospitalizations and does not encompass the entire spectrum of youth mental health–related events, such as outpatient and emergency department visits.

## Methods

We conducted a retrospective cohort study using routinely collected administrative health data of hospital admissions between 1 April 2018 and 5 March 2022. This allowed us to capture mental health–related inpatient hospitalizations for approximately 2years before and 2 years after the start of the COVID-19 pandemic (set as 15 March 2020). Our analysis included individuals aged 10 to 17 years with a valid Medicare health card number at the time of hospitalization. 

Data were obtained from the Canadian Institute for Health Information’s Discharge Abstract Database (DAD) housed at the Public Health Agency of Canada. The Discharge Abstract Database is a comprehensive national (excluding Quebec) database that has been capturing hospital discharge abstracts for acute, chronic and rehabilitative care since 1988.^16^ The use of these administrative data is authorized under section 44 of Ontario’s *Personal Health Information Protection Act*, which does not require review by a research ethics board.^17^

***Outcomes***

The primary outcome was an inpatient hospitalization for a diagnosis related to mental health and addiction, including any diagnosis of deliberate self-harm or substance use-related disorder. The secondary outcomes were: (1) inpatient hospitalization with a diagnosis of deliberate self-harm; and (2) inpatient hospitalization with a diagnosis of substance use disorder (including poisoning codes).^18^ The code algorithms used were based on Canadian Institute for Health Information (CIHI) definitions.^18^-^20^ Unless otherwise stated, the primary and secondary outcomes were summarized as number of weekly events per 100 000 persons. An event constitutes one episode of care, which refers to all continuous inpatient hospitalizations (including transfers within or between facilities) as defined by the Canadian Institute for Health Information (CIHI). Transfers were considered one episode of care if they occurred less than 7hours after discharge, regardless of whether there was a transfer code, or less than 12 hours after discharge if at least one of the visits had a transfer code.^21^ Two or more separate events from the same individual are counted as multiple events rather than a single event. We estimated annual population counts of youth aged 10 to 17 years using Statistics Canada’s annual population estimates, broken down by sex and region.^22^ The same estimated population was used for the entire calendar year; this assumed a constant annual population growth, which appeared to be supported by the data. Age was calculated on the date of admission and province was determined based on the issuing health card.

***Statistical analysis***

We conducted an interrupted time-series analysis to assess the impact of the pandemic on trends and rates of mental health-related hospitalizations before and after 15 March 2020. We used segmented linear regression analysis on the weekly hospitalization rates in all of Canada (except Quebec), as well as each region (i.e. province/territory) spanning 104 weeks (2 years) before the pandemic. 

We expected trends during the early weeks of the pandemic to differ from those during the overall pandemic as individuals and institutions adjusted to changes in service delivery and diagnosis of mental health-related events. Hence, we examined two interruptions: (1) early pandemic period spanning 16 weeks, and (2) the rest of the pandemic period spanning 89 weeks. We refer to the pre-pandemic period as T0 (1 April 2018 to 14 March 2020), the early pandemic period as T1 (15 March 2020 to 5 July 2020) and the later pandemic period as T2 (6 July 2020 to 5 March 2022). We chose 5 July 2020 as the end of T1 as some American studies had shown that hospitalization rates returned to expected rates between late June and mid July.^23^ Furthermore, this was also the first period where most provinces and territories relaxed some public health measures.^24^

We combined data from the territories (Yukon, Northwest Territories and Nunavut) and the Atlantic provinces (New Brunswick, Prince Edward Island, Nova Scotia, and Newfoundland and Labrador) due to a small number of events for some analyses. To ensure that patterns within different youth age groups were assessed, we also stratified the Canada-wide results by age: 10 to 14 years and 15 to 17 years.

The model included coefficients for T0 (102 weeks), T1 (16 weeks) and T2 (89 weeks). All analyses considered autocorrelation with lag one and adjusted for seasonality by adding a covariate for each month (i.e. January to December).^25^ We confirmed that model assumptions of homoscedasticity, linearity and normality (assessed graphically) were met for all models. We tested for autocorrelation using the Durbin–Watson statistic and used the Cook D statistic to ensure there were no influential data points.^25^ All analyses were conducted using SAS version 9.4 (SAS Institute Inc., Cary, NC, US) with a modified version of an interrupted time-series SAS macro.^26^

For each study group, we presented the baseline hospitalization rate per 100 000 as well as the trend (stable, statistically significantly increasing, statistically significantly decreasing) during three periods (T0, T1 and T2).

## Results

There were 73 907 mental health and addiction–related hospitalizations of youth across Canada (except Quebec) during our study period (209 weeks). The mean age was 14.8 years and the median was 15years (IQR = 2). Females had higher overall mental health and addiction–related hospitalization rates than males across all provinces in an approximately 2:1 ratio (Table 1).

**Table 1:** Mental health and addiction-related hospitalizations of youth (10–17 years) by geographic region and by sex, Canada (except Quebec), 1 April 2018 to 5 March 2022

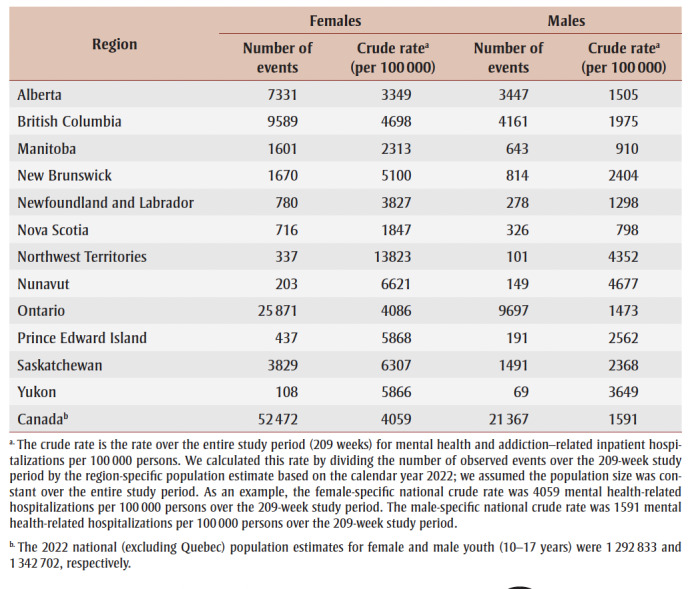

***Changes in mental health and addiction–related inpatient hospitalization***

**Figure 1:**
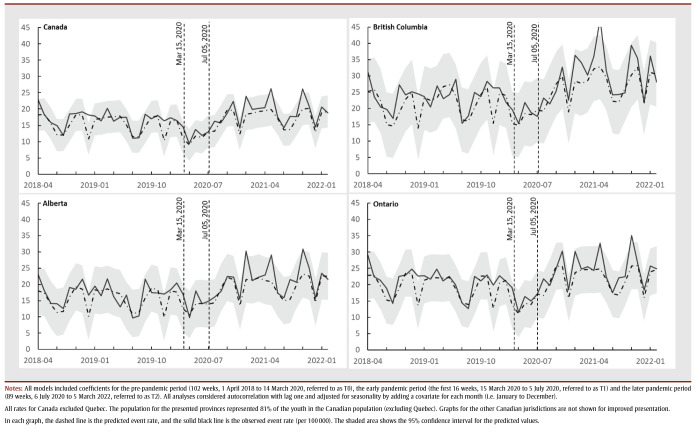

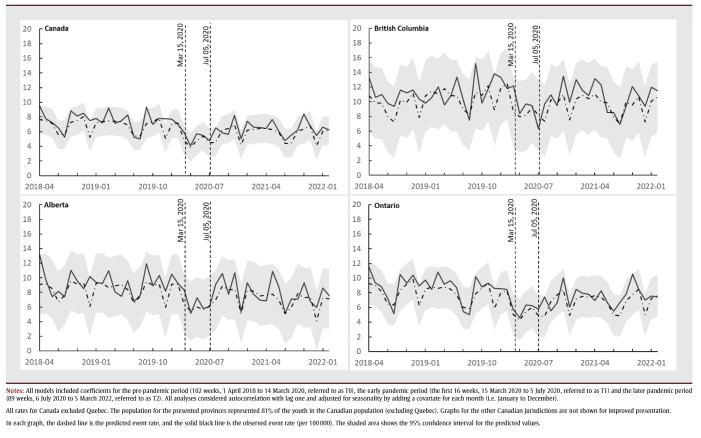
Weekly hospitalization rate (per 100 000) with mental health and addiction diagnosis of female youth (10–17 years) in Canada (except Quebec) and British Columbia, Alberta and Ontario, April 2018 to March 2022 - Weekly hospitalization rate (per 100 000) with mental health and addiction diagnosis of male youth (10–17 years) in Canada (except Quebec) and in British Columbia, Alberta and Ontario, April 2018 to March 2022

The pre-pandemic (T0) provincial/territorial baseline rates for mental health and addiction–related hospitalizations ranged from 6.27 (in Manitoba) to 85.59 (in the territories) weekly events per 100000 persons. These rates were stable for all provinces and territories during this period (Figures 1a and 1b). In general, hospitalization rates for females were higher than for males. Adjusted for seasonality, the national hospitalization rate during T0 was 12.82 (95% CI: 12.14 to 13.50) weekly events per 100 000 persons ( Table 2). 

**Table 2:** Average Canada-wide weekly rates per 100 000 persons of hospitalizations with mental health and addiction, self-harm and substance disorders, all youth, by sex, and by age group (10–14 years and 15–17 years), Canada (except Quebec), April 2018–March 2022

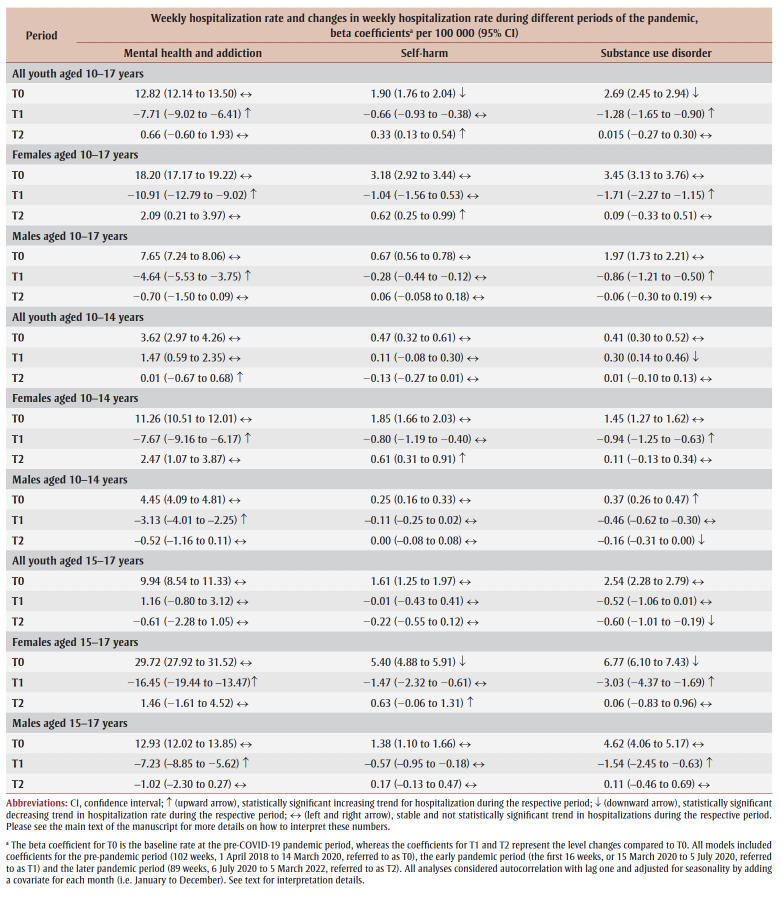

During T1, the average weekly hospitalization rate dropped from 12.82 (95% CI: 12.14 to 13.50) by 7.71 (95% CI: 6.41 to 9.02) events per 100 000 persons, compared to the baseline period. The level change in hospitalization rate dropped to 5.11 (95% CI: 3.80 to 6.41), equating to a 60% (95% CI: 50% to 70%) percentage decrease compared to T0. We also observed a statistically significant increasing trend in the weekly rates per 100000 persons as the early pandemic progressed. This rate increase was also statistically significant among males in British Columbia, Ontario and Manitoba and females in British Columbia, Ontario, Alberta, Saskatchewan and Manitoba, from highest to lowest rate of increase per sex (Figures 1a and 1b; graphical data not shown for Saskatchewan and Manitoba).

During T2, the national weekly hospitalization rate was similar to T0 for the entire cohort and for males. The female national hospitalization rate increased from 18.20 (95% CI: 17.17 to 19.22) events per 100 000 persons in T0 to 20.29 (95% CI: 18.41 to 22.17) events per 100 000 persons, equating to an 11% (95% CI: 1% to 21.8%) increase (Figures 1a and 1b). There was no statistically significant change in the trend for hospitalization rate during T2 for either males or females at the national or provincial/territorial levels. There were also no notable differences in the national trend for youth aged 10 to 14 years and 15 to 17years compared to the trend for youth overall.

***Changes in self-harm–related inpatient hospitalizations***

**Figure 2:**
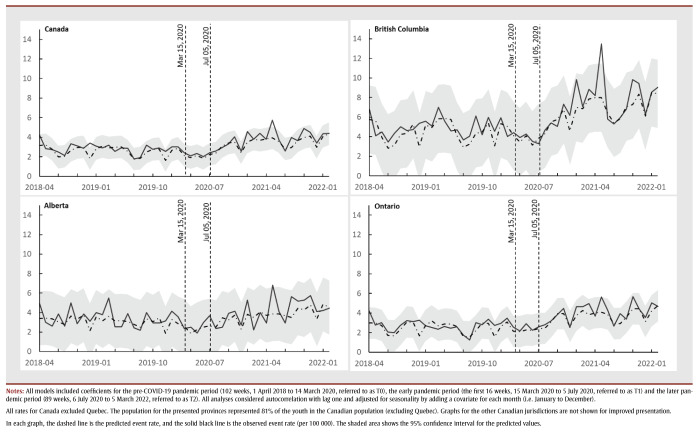

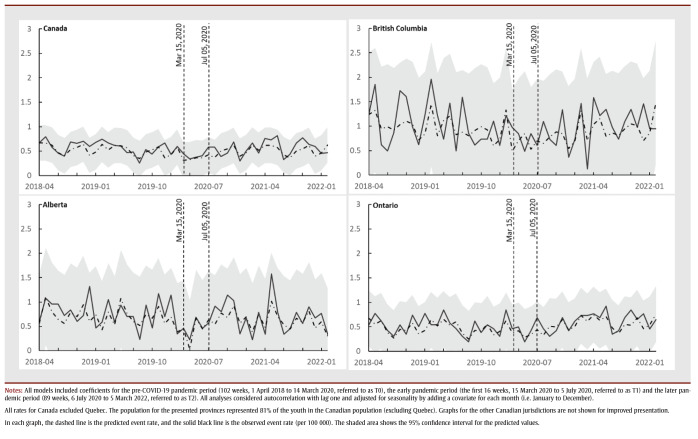
Weekly hospitalization rate (per 100 000) with self-harm diagnosis of female youth (10–17 years) in Canada (except Quebec) and in British Columbia, Alberta and Ontario, April 2018 to March 2022 - Weekly hospitalization rates (per 100 000) with self-harm diagnosis of male youth (10–17 years) in Canada (except Quebec) and in British Columbia, Alberta and Ontario, April 2018 to March 2022

The self-harm–related hospitalization rate during T0 was higher for females than for males for all provinces; the national rate for the entire cohort was 1.90 (95% CI: 1.76 to 2.04) weekly events per 100 000 persons, and rates remained stable during this time (Table 2 ). During T1, the rates also remained stable for the entire cohort. There was a statistically significant increasing trend in the national rate of hospitalizations of females during T2, as well as for British Columbia, Ontario, Alberta and the Atlantic provinces, from highest to lowest rate of increase (Figures 2a and 2b; graphical data not shown for Atlantic provinces); we observed no statistically significant change in trend for males. At the national level, the level rate for females increased from 3.18 (95% CI: 2.92 to 3.44) in T0 to 3.79 (95% CI: 3.42 to 4.16) events per 100 000 persons in T2, equating to a 19% (95% CI: 7% to 31%) increase. We also found a statistically significant increasing trend in rates for females aged 10 to 14years and 15 to 17 years. However, the level change was only statistically significant for females aged 10 to 14 years; the rate increased from 1.85 (95% CI: 1.66 to 2.03) in T0 to 2.46 (95% CI: 2.16 to 2.76) events per 100 000 persons in T2, equating to a 32% (95% CI: 16% to 49%) increase.

***Changes in substance use disorder–related inpatient hospitalizations***

**Figure 3:**
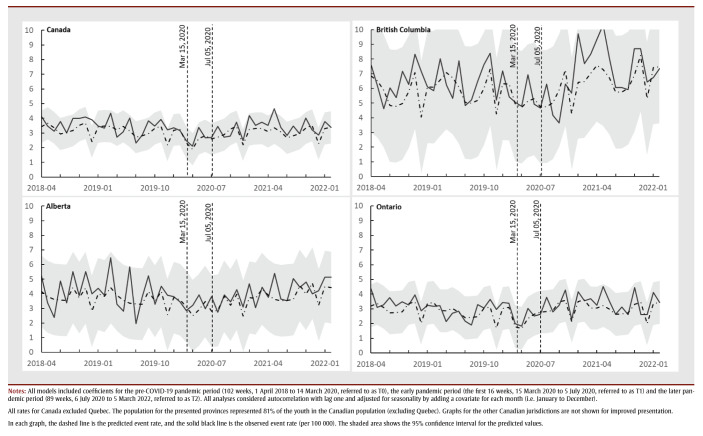

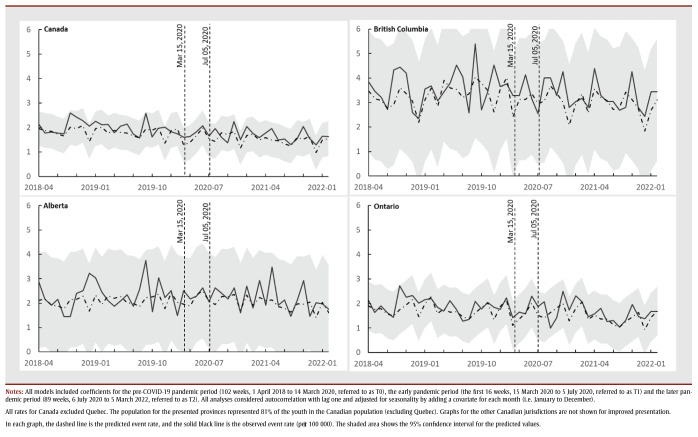
Weekly hospitalization rate (per 100 000) with substance use disorder diagnosis of female youth (10–17 years) in Canada (except Quebec) and in British Columbia, Alberta and Ontario, April 2018 to March 2022 - Weekly hospitalization rate (per 100 000) with substance use disorder diagnosis of male youth (10–17 years) in Canada (except Quebec) and in British Columbia, Alberta and Ontario, April 2018 to March 2022

The substance disorder–related hospitalization rate during T0 was higher for females than males across all regions. At T0, the national substance disorder–related hospitalization rate was 2.69 (95% CI: 2.45 to 2.94) events per 100 000 persons (Table 2). During T1, the substance disorder–related hospitalization rate decreased to 1.41 (95% CI: 1.04 to 1.79) events per 100 000 persons, equating to a 50% decrease (95% CI: 39% to 66%). There was a statistically significant decrease in the national trend for substance disorder–related hospitalizations during T1 for both sexes and among females. There were no significant level changes or trends in the rates of hospitalizations with substance disorder among both males and females across all regions during T2 (Figures 3a and 3b). We observed similar results for youth aged 10 to 14years and those aged 15 to 17 years.

## Discussion

We conducted a Canada-wide analysis to understand rate changes in mental health and addiction–related hospitalizations among youth during different phases of the COVID-19 pandemic. We found regional variations, with the territories showing the highest hospitalization rates before and during the pandemic;^16^ this finding may be attributed to a complex interplay of factors, one of which could be limited outpatient resources in the territories, resulting in a greater reliance on inpatient hospitalizations. While there was no general increase in the mental health and addiction–related hospitalization rate, we observed a concerning rise in the self-harm–related hospitalization rate among females nationally and within most provinces.

Some Canadian studies have shown an increase in mental health–related outpatient and emergency department visits, especially among young females.^2^,^11^,^16^,^27^-^29^ However, our study did not find a corresponding increase in the mental health and addiction–related inpatient hospitalization rate.^30^ We also did not find significant changes in substance disorders, which is in line with past findings. Presentations to the emergency department for substance abuse by youth decreased during the pandemic, apart from opioid-related emergency department visits.^31^,^32^ While there is evidence that there might have been an increase in the frequency of alcohol and cannabis use by youth during the pandemic, our results and past findings suggest this does not lead to an increase in visits to the hospitals and emergency departments.^33^,^34^

The rise in the rate of self-harm–related hospitalizations among female youth is alarming and calls for attention; evidence suggests it may have started before the pandemic.^35^ This trend should guide policy makers and clinicians in allocating resources and shaping public health strategies to meet the mental health needs of this demographic. Our study also raises questions about whether the observed sex differences in hospitalization rates, particularly for self-harm and, to a lesser extent, substance disorders, could be related to systemic biases in clinical decision-making and/or gender differences in help-seeking behaviors.^29^,^36^,^37^

Our findings should not be interpreted to mean that mental health and addiction conditions have not increased among young people in Canada. The increase in self-reported mental health conditions and unmet needs of mental health care post-pandemic among youth continues to be corroborated by Statistics Canada and other large Canadian surveys.^5^,^38^ Further, our findings may only pertain to the most severe mental health and addiction–related events, and non-hospitalized mental health and addiction events may have continued to increase. In other words, in the majority of the provinces, mental health and addiction events are not taking up inpatient hospitalizations.

***Strengths and limitations***

The strength of our study lies in its comprehensive coverage of included Canadian provinces and territories. However, our study also has several limitations. First, we used administrative data and diagnostic coding algorithms with high specificity but moderate to low sensitivity, potentially underestimating mental health and addiction–related hospitalization rates.^39^ Second, the exclusion of Quebec limits the study’s nationwide applicability. Third, the pandemic strained health care systems, altering hospital visit protocols and health care worker availability, which may have influenced our observed rates. Fourth, the observed trends, especially during the early pandemic period (T1), could be attributed to delayed health care–seeking behaviours for mental health and addiction–related issues due to individuals’ reluctance to interact with the health care system during the pandemic, as observed in other health care contexts.^40^-^42^ Fifth, underlying systemic biases in clinical decision-making may also affect hospital admission rates. For example, if health care providers are more inclined to admit females than males for mental health conditions, this could skew the observed sex-specific hospitalization rates.^10^,^43^,^44^ Lastly, our findings might be influenced by unaccounted factors such as neighborhood-level socioeconomic status, racial/ethnic backgrounds and access to either the means or opportunity to self-harm or use substances.

## Conclusion

While our study indicated no significant increase in overall mental health and addiction–related hospitalizations among Canadian youth during the pandemic, the rise in self-harm hospitalization rate among females warrants focused attention. These findings highlight the need for ongoing monitoring and research to better understand and address the mental health challenges faced by Canadian youth.

## Acknowledgements

The authors would like to acknowledge the Canadian Institute for Health Information for collecting and providing the data used in this manuscript. The authors also thank Li Liu and Wendy Thompson for initial feedback on the proposal. 

## Conflicts of interest

The authors have no conflicts of interest.

Justin J. Lang is one of this journal’s Associate Scientific Editors, but has recused himself from the review process for this article.

## Authors’ contributions and statement

CD: Conceptualization, data curation, formal analysis, investigation, methodology, project administration, validation, visualization, writing original draft, writing review and editing

AAA: conceptualization, data curation, formal analysis, investigation, funding acquisition, methodology, project administration, validation, visualization, supervision, writing – original draft, writing – review and editing

EC: Conceptualization, data curation, methodology, project administration, writing – review and editing

CO: Conceptualization, formal analysis, methodology, project administration, writing – review and editing

NA: Conceptualization, investigation, project administration, writing – review and editing

JJL: Conceptualization, investigation, project administration, writing – review and editing

IC: Conceptualization, investigation, project administration, writing – review and editing

MW: Conceptualization, project administration, writing – review and editing

RE: Conceptualization, data curation, funding acquisition, investigation, resources, supervision, project administration, writing – review and editing

The content and views expressed in this article are those of the authors and do not necessarily reflect those of the Government of Canada.
